# Minimally invasive surgery: a concept already incorporated

**DOI:** 10.1590/S1516-31802013000100015

**Published:** 2013-04-01

**Authors:** Alessandro Wasum Mariani, Paulo Manuel Pêgo-Fernandes

**Affiliations:** I IMD, PhD. Thoracic Surgeon, Instituto do Coração (InCor), Hospital das Clínicas (HC), Faculdade de Medicina da Universidade de São Paulo (FMUSP), São Paulo, Brazil.; II MD, PhD. Associate Professor, Discipline of Thoracic Surgery, Instituto do Coração (InCor), Hospital das Clínicas (HC), Faculdade de Medicina da Universidade de São Paulo (FMUSP), São Paulo, Brazil.

Minimally invasive procedures can be defined as those that can be performed by entering the body with the minimum damage to the entrance, which may be the skin, a body cavity or an anatomical opening. In contrast with the ancient aphorism “great surgeons, great incisions”, the current trend regarding incisions is much closer to “the less, the better”.

The benefits from reducing injuries to the access route that are most advocated are the following: reduction of postoperative pain, reduction of bleeding, reduction of the inflammatory response to the trauma and a better esthetic result.^1^ However, the degree of proof of these benefits varies according to the specialty, and even between specific procedures.

Examples of procedures that are said to be minimally invasive encompass a variety of fields, namely: in abdominal surgery, videolaparoscopy; in orthopedic surgery, arthroscopy; in thoracic surgery, videothoracoscopy; in cardiology, interventionist cardiology; in vascular surgery, interventionist vascular procedures; in radiology, radio-intervention; among others. This is without taking into account the advent of robotic surgery, which has gained space in all fields of surgery.

However, all the new techniques developed within the sphere of minimally invasive surgery go beyond the basic concept of simply reducing the size of the incision. These advances and discoveries have unequivocally contributed towards accelerating the overall development of surgery, and even what is known as “conventional” or “open” surgery.

Materials that were initially developed for video surgery are sometimes used in “open” cases to shorten the duration of the operation, thus providing a real benefit. Many “hybrid” procedures can also be cited as examples. Among these procedures, one that is well known is aortic aneurysm correction performed by associating open surgery for the proximal portion and placement of an endoprosthesis in the distal portion.^2^

One very interesting issue is the way in which concepts and techniques acquired through experience of video surgery end up influencing or modifying how to perform open surgical cases. One good example is how the stump is treated in appendectomy cases: after gaining experience of video-guided stapling,^3^ many surgeons ceased to routinely perform the classical “tobacco pouch” technique, which was a very common and well-established concept.

In thoracic surgery, the tactic known as the fissureless technique^4^, which is used for accessing the pulmonary hilum to treat the fissure, is being incorporated by many thoracic surgeons, who perform videothorascopic lobectomy even when they operate on open cases.

In surgical treatment for inguinal hernias, there have been interesting experiences using video surgery, with regard to the way in which the mesh is fixed.^5^

In relation to cardiovascular surgery, there has been an important discussion about diminishing not only the trauma of the incision but also the trauma caused by use of extracorporeal circulation. Along these lines, many myocardial revascularization procedures have been performed with the heart beating through the aid of special devices known as cardiac stabilizers, thereby suppressing the need for extracorporeal circulation.^6^ Another trend in cardiac surgery without extracorporeal circulation consists of using transapical implantation of a valved endoprosthesis into the aortic position.^7^

With regard to perioperative management, minimally invasive surgery has helped to establish the concept of avoiding blood transfusion whenever possible. A variety of studies in the literature have demonstrated that avoiding transfusion has major benefits.^8^ However, for this to be done safely, it is very important to have cooperation between surgeons (to reduce bleeding), anesthetists (to modulate the fluid supply) and intensive care specialists (to improve the postoperative monitoring and management).

The discussion relating to minimally invasive surgery ultimately always brings up the issue of cost. It is a fact that use of higher technology raises expenditure. However, in many cases, corresponding savings can be expected, due to the shorter hospital stay, faster recovery and lower complication rates. Nonetheless, such savings may not be real for all minimally invasive procedures. Repeating, all technological gains generate costs and this needs to be discussed specifically for each field of medicine, taking into account the scientific proof of the benefit, cost effectiveness, availability of resources and technical knowledge among the people involved, among other factors.

These are some of the reasons for believing that modern surgeons should not have borders or make distinctions between what is known as conventional surgery and as minimally invasive procedures. Surgeons should be capable of performing any form of treatment that is pertinent to their specialty, while always adapting the indication and implementation of each procedure to each patient’s real needs and conditions. In this manner, not only a “minimally invasive” procedure but also a “maximally resolutive” and appropriate procedure is generated.
